# Acetone Sensing Properties and Mechanism of Rh-Loaded WO_3_ Nanosheets

**DOI:** 10.3389/fchem.2018.00385

**Published:** 2018-09-11

**Authors:** Zhilei Qiu, Zhongqiu Hua, Yan Li, Mengjun Wang, Dan Huang, Chen Tian, Chensheng Zhang, Xuemin Tian, Erping Li

**Affiliations:** Tianjin Key Laboratory of Electronic Materials and Devices, School of Electronics and Information Engineering, Hebei University of Technology, Tianjin, China; Key Laboratory of Micro-Nano Electronics and Smart System of Zhejiang Province, Department of Information Science & Electronic Engineering, Zhejiang University, Hangzhou, China

**Keywords:** WO_3_, Rh, acetone, surface modification, gas sensors

## Abstract

WO_3_ nanosheets was prepared by an acidification method and the Rh catalyst was dispersed on the surface of the nanosheets with a wet impregnation method. The morphology of pristine WO_3_ and Rh modified WO_3_ nanosheets and their responses to acetone gas were studied. According to oxygen adsorption combined with TPR results, the sensing and sensitization mechanism of acetone were discussed. It was found that no visible changes in nanostructures or morphologies were observed in WO_3_ nanosheets with Rh, however, the sensor resistance and sensor response were greatly promoted. The basic sensitization mechanism could be caused by the electronic interaction between oxidized Rh and WO_3_ surface.

## Introduction

Acetone gas is closely related to people with diabetes. Medical research has shown that there is a significant difference of acetone concentration in the breath for diabetics and healthy people, the former being higher than 1.8 ppm and the latter being below 0.8 ppm (Owen et al., 1982; Natale et al., 2014). Therefore, through the quantitative detection of the acetone concentration in human exhaled gases, it could achieve the purpose of diagnosis and monitoring to the disease condition. Metal oxide semiconductors (MOS) have been widely reported for gas sensors with the significant advantages, such as low cost, simple process and small size (Hübner et al., 2010; Choi et al., 2014). Tungsten trioxide (WO_3_) as an n-type semiconductor has become a research hotspot in the detection of VOC gases in recent years (Kanda and Maekawa, 2005; Kadir et al., 2015; Li et al., 2017). The adsorption and reaction of VOC gas on WO_3_ surface could change the semiconductor resistance, so the gas response can be improved by adding highly efficient catalytic elements. The introduction of ruthenium (Ru) and silicon (Si) improve the sensitivity of WO_3_ to acetone (Righettoni et al., 2010; Li et al., 2018). Further, Rh is known as a highly efficient catalyst to the catalytic reaction of acetone gas (Houtman and Barteau, 1991). It has been reported that Rh loaded SnO_2_ and In_2_O_3_ significantly improve the response of acetone (Kim et al., 2011; Kou et al., 2018). Therefore, this highly efficient catalyst could be also loaded onto WO_3_ surface to increase the response to acetone. In this study, the Rh element was uniformly loaded onto the surface of WO_3_ nanosheets based on an impregnation approach. This method has been frequently used in our previous work (Li et al., 2018). The experimental results show that the Rh nanoparticles can significantly enhance the response of WO_3_ nanosheets to acetone without changing the surface morphology of WO_3_ nanosheets. The basic sensitization mechanism of Rh was also analyzed based on experimental results.

## Experimental

WO_3_ nanosheets was obtained by dropping Na_2_WO_4_ solution into H_2_SO_4_ solution (Kida et al., 2009). Aqueous solution of RhCl_3_ was impregnated with WO_3_ (Rh-WO_3_) powders and formed a suspension slurry, which was washed by distilled water and dried. Subsequently, the powders were annealed at 500°C in air. Sensor devices were made by the screen-printing technique. The crystal structures were measured by X-ray diffractometer (XRD; D8FOCUS, Germany). The morphology of sample was analyzed using scanning electron microscopy (FE-SEM; Nova Nano SEM 450, FEI, U. S). Nanosheets were also characterized by a transmission electron microscopy (TEM; Tecnai-F20, FEI, U.S). Energy spectrum analysis of materials uses X-ray photoelectron spectroscopy (XPS, Thermo escalab 250Xi, U. S). The catalyst activity was characterized by H_2_ temperature programmed reduction (H_2_-TPR; TP-5076, China). The experimental procedure of the TPR was descripted in Figure S1. Gas sensing tests were carried out by a conventional gas flow apparatus (see Figure S2). The gas sample was kept at a constant flow rate of 100 ml/min by mass flow controllers (MFC). The humidity of gas was <20 ppm and temperature of the chamber was about 50°C. The sensor response (*S*) was defined as *S* = *R*_*a*_/*R*_*g*_, where *R*_*a*_ and *R*_*g*_ are the sensor resistance in air and in the presence of target gases.

## Results and discussion

**Figure 1:**
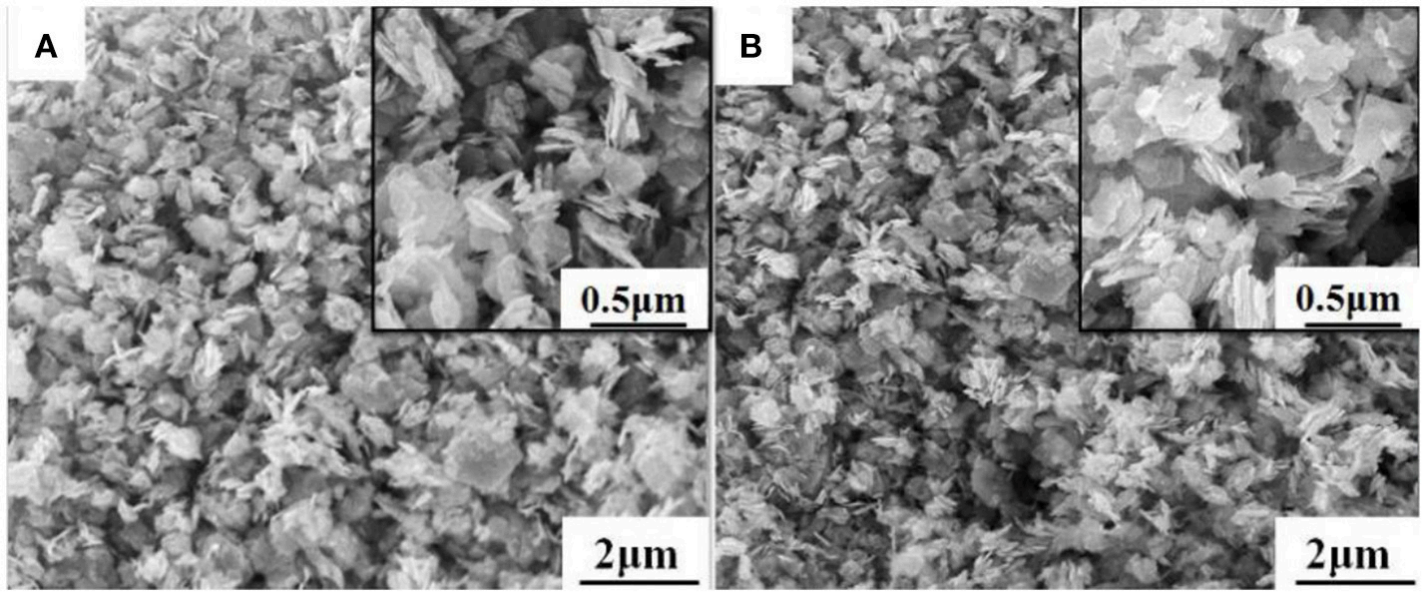
SEM images of **(A)** pure WO_3_ nanosheets and **(B)** 1wt%Rh-WO_3_ nanosheets.

**Figure 2:**
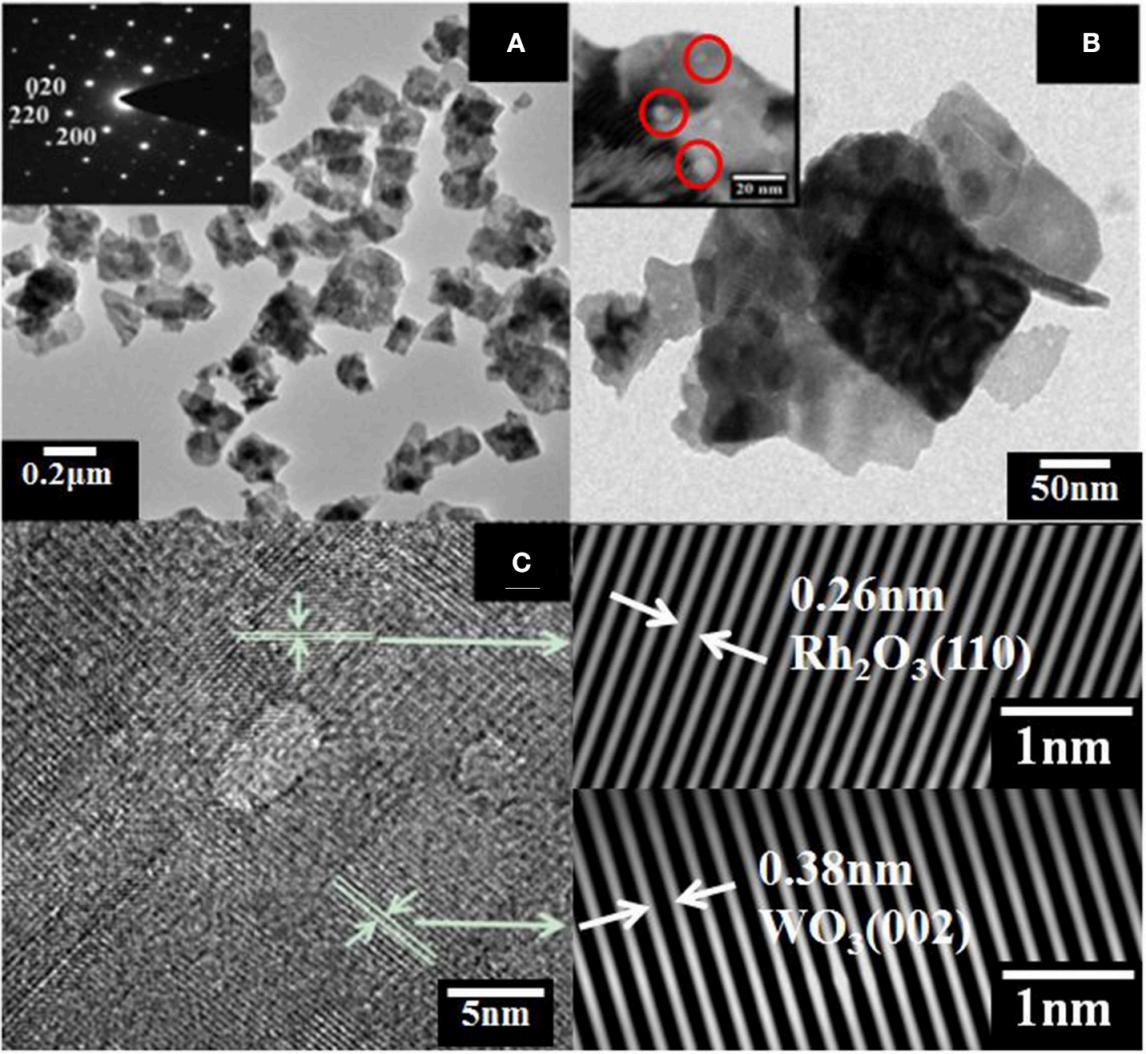
TEM images of **(A)** WO_3_ nanosheets insert with selected area diffraction (SAD) pattern, **(B)** 1wt%Rh-WO_3_ nanosheets, **(C)** HRTEM image of 1wt%Rh-WO_3_ nanosheets and SAD pattern of Rh_2_O_3_ and WO_3_.

The morphology of WO_3_ nanosheets was characterized by SEM and TEM. Figures 1A,B show SEM images of pristine and 1wt.% Rh-WO_3_ nanosheets. One can see that the sample powders consisted of a large amount of nanoparticles with a lateral size from dozens to several hundred nanometers. According to SEM images, there were no visible changes observed in pristine WO_3_ and Rh modified one. For the results of specific surface area, pristine WO_3_ was ~12 m^2^/g and 1wt. %Rh-WO_3_ is about 13 m^2^/g, which indicts no significant change. Figure 2 shows TEM images of the pure WO_3_ and 1wt.%Rh-WO_3_ nanosheets. It was obvious that the sample powder is actually composed of highly irregular plate-like nanosheets. The insert image of Figure 2A presents a selected area diffraction (SAD) pattern of pristine WO_3_ nanosheets, suggesting that the nanosheets have a good crystal quality. In addition, some white particles with dozens of nm in size were observed in WO_3_ surface, as shown in Figure 2B. With the help of SAD in Figure 2C, these particles were identified as Rh_2_O_3_ with lattice spacing of 0.26 nm, corresponding to the (110) plane (JCPDS: 25-0707). It was thought that these large particles of Rh_2_O_3_ could be due to the aggregation of Rh during washing and drying process, which were not effectively removed during the washing process. While the lattice spacing of 0.38 nm in the HRTEM image was belong to monoclinic WO_3_ (JCPDS: 43-1035), which was in a good agreement of XRD results (in Figure S3).

**Figure 3:**
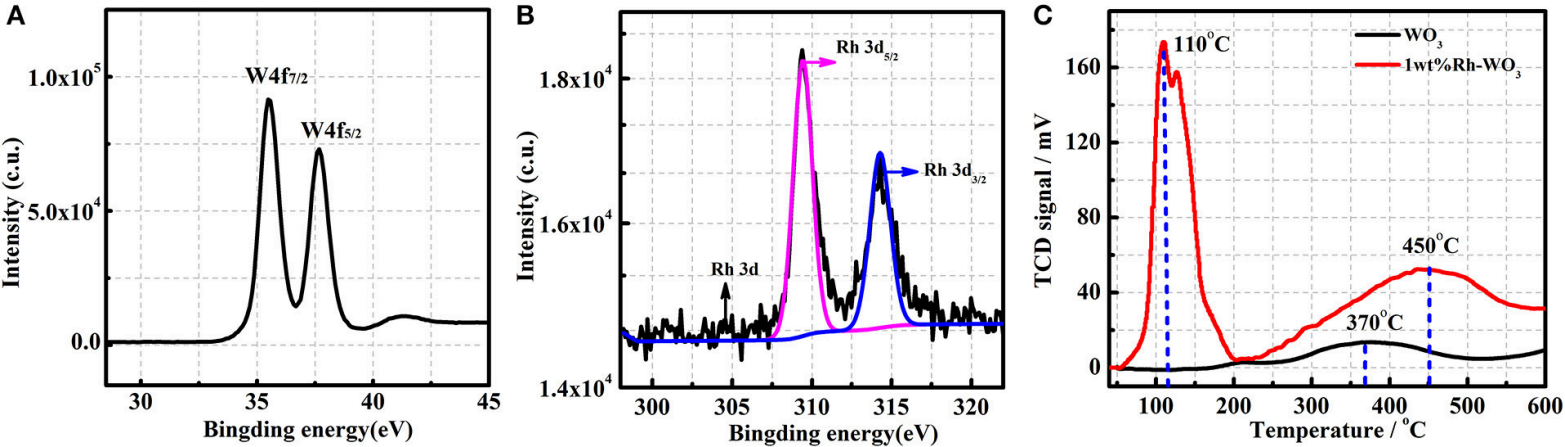
The XPS of 1wt%Rh-WO_3_: **(A)** W4f, **(B)** Rh3d, and **(C)** the H_2_-TPR patterns of pure WO_3_ and 1wt%Rh-WO_3_.

The chemical state of Rh on WO_3_ surface was also analyzed by XPS. Figure 3A presents the XPS spectra of W, detection results of binding energy for W4f_7/2_ and W4f_5/2_ being 35.7eV and 37.9eV, respectively, which is in good agreement with W^6+^ (Dupin et al., 2000). Figure 3B shows the XPS spectra of Rh3d obtained from 1wt%Rh-WO_3_. Among them, the Rh2d_5/2_ peaked at 309.45eV is a typical oxide centered on Rh^3+^. In addition, the Rh2d_3/2_ located at 314.5eV is also an oxide centered on Rh^3+^ (Kim et al., 2011). Thus, it could be concluded that Rh was present as an oxidized state of Rh_2_O_3_ on WO_3_ surface. Additional with XPS results, the oxidized state of Rh could be also evidenced by H_2_-TPR. Figure 3C shows the H_2_-TPR results of pristine and 1wt%Rh-WO_3_ nanosheets. As expected, there was one weak peak around 370°C observed in pristine WO_3_ nanosheets indicating a weak consumption of H_2_, which may be due to the weak reduction behavior of WO_3_ surface at a high temperature (Li et al., 2018). In contrast, large consumptions of H_2_ were observed in 1wt%Rh-WO_3_, suggesting a strong reduction behavior. There were two overlapped peaks of H_2_ consumption at a low temperature around 110°C and the intensities of peaks were relatively high. It was believed that the consumption of H_2_ observed at low temperatures could be due to the reduction of Rh_2_O_3_ and peaks located different temperature may be ascribed to different dispersion states of Rh species. There was a broad but weak peak of H_2_ consumption at around 450°C, which could be attributable to the weak reduction of WO_3_ surface, i.e., surface lattice oxygen (O_L_) reacting with H_2_ at a high temperature. The reduction behavior of Rh-WO_3_ was much stronger than pristine WO_3_ indicating that the reactivity of lattice oxygens is slightly promoted by Rh_2_O_3_ on the surface. We can see significant differences, comparing this reduction behavior with our previous study of Pt-WO_3_ nanosheets (in Figure S4). At low temperature, Pt-WO_3_ produces a negative peak of H_2_ desorption. Based on the results of TPR and the resistance behavior under P_O2_, it is concluded that the main sensitization of Pt-WO_3_ may be caused by redox of Pt. nanoparticles (Li et al., 2017). This phenomenon of Rh may cause different sensitization mechanisms.

**Figure 4:**
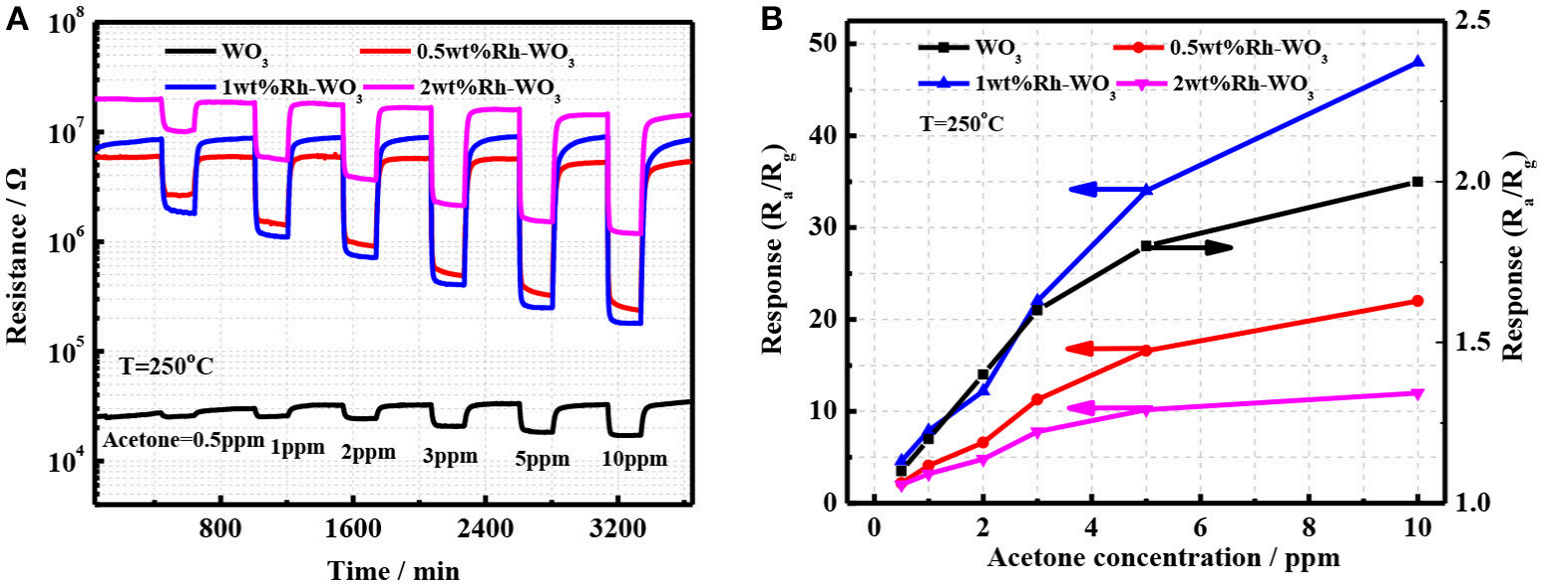
**(A)** The transient response of four different sensor devices and **(B)** the sensor response as a function of acetone concentration.

The sensing properties of pristineWO_3_ and Rh-WO_3_ nanosheets were characterized with acetone ranging from 0.5 to 10 ppm. Figure 4A shows the time dependence of sensor resistance. It was worth noting that the introduction of Rh greatly increased the sensor resistance of WO_3_. For 2wt.% Rh-WO_3_, the sensor resistance was almost three orders of pristine one. This indicated a strong electronic interaction between Rh_2_O_3_ and WO_3_ surface, forming the well-known P-N junction or fermi-level control sensitization mechanism. Due to the electronic junction of Rh_2_O_3_ with WO_3_, the sensors response and responding speed were significantly promoted. Figure 4B shows the calibration line of sensors resistance with concentration of acetone at an operation temperature of 250°C. It was found that sensor based on 1wt.%Rh-WO_3_ also responded to 0.5 ppm acetone. One can see that sensor response was increased by 3 times compared with the neat WO_3_. However, an excess of Rh did not effectively to promote the sensor response. This observation was in conflict with the great enhancement in sensor resistance. In order to explain the reduction in sensor response for 2wt.%Rh-WO_3_, there were two factors should be considered. Firstly, an excessive amount of Rh could lead to agglomeration of Rh_2_O_3_ and poor dispersion on the surface of WO_3_ nanoparticles. Consequently, some electronic interaction of Rh_2_O_3_ with WO_3_ leading to the high resistance were not effective to the sensitization. Secondly, with increasing the amount of Rh the surface activity of WO_3_ could be enhanced and then leaded to a catalytic reaction of acetone, which inhibit the diffusion of acetone molecule into inside of sensor films. As a result, the sensor response was reduced by a high loading amount of Rh. This reduction in sensor response could be also observed when increasing operation temperatures. This was evidenced by the strong dependence of sensor response on the operation temperatures for Rh-WO_3_ as shown in Figure S5a. It was thought that increasing the operating temperature leaded to an enhancement in catalytic activity, which reduces the gas diffusion and sensor response to acetone. When operating at a temperature larger than 250°C, one can note the sensor response greatly decreased with temperatures. It was also found that sensors resistance also obviously decreased with temperature, as shown in Figure S5b. For pristine WO_3_, the sensor response did not change significantly with temperature and exhibited a lower response at different temperatures. This poor response is associated with a weaker oxygen adsorption on WO_3_ surface (Zeng et al., 2017). At the same time, the stability of the sensor was also evaluated as shown in Figures S5c,d. It can be seen that Rh-WO_3_ nanoparticles can work for a long time at 350°C and has favorable response recovery performance.

**Figure 5:**
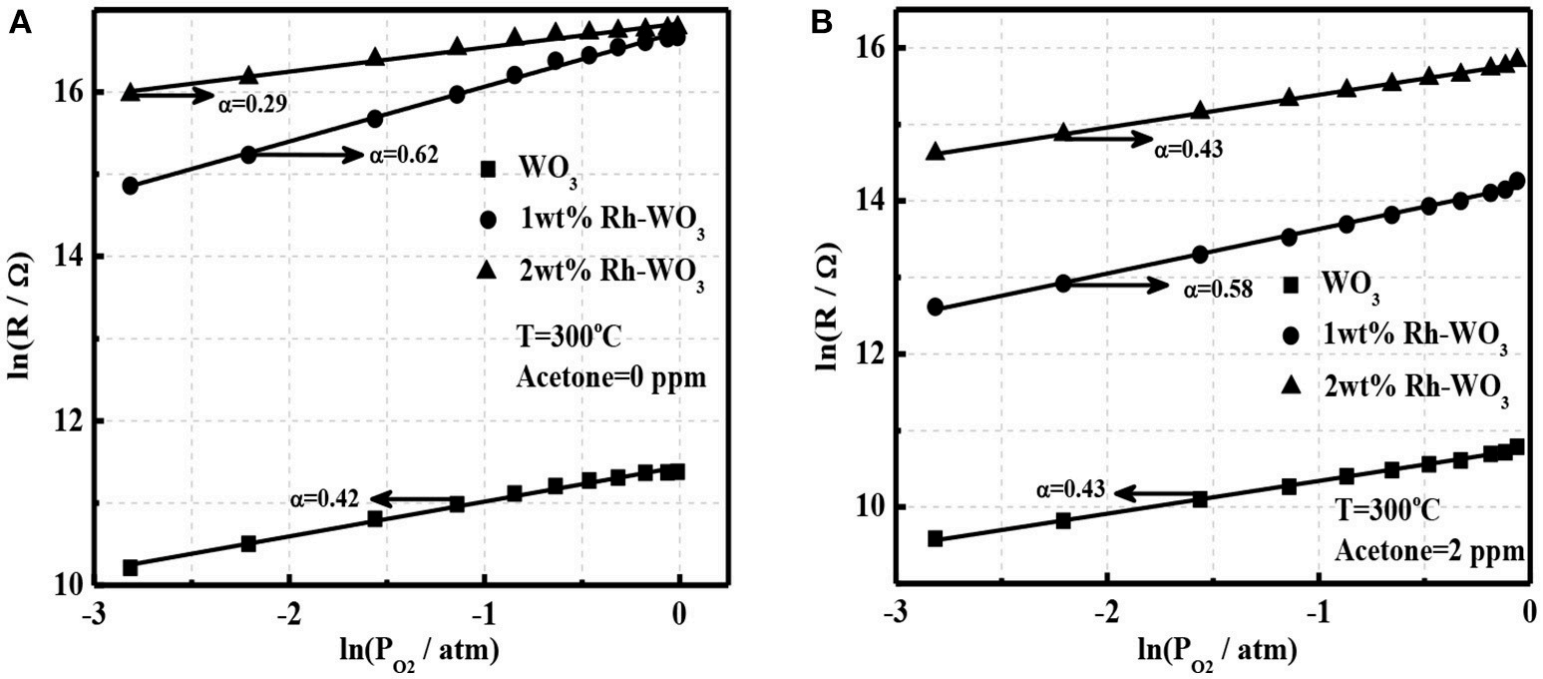
The power-law response to oxygen for pristine WO_3_ and Rh-WO_3_ **(A)** in the absence of acetone and **(B)** in the presence of 2 ppm acetone at 300°C.

It is well-known that oxygen adsorption in the form of ${\text{O}}_{2}^{-}$, O^−^, or O^2−^ on the surface serves as the receptor function and determines the sensing ability and mechanism of MOS gas sensors (Hua et al., 2018a). In order to explore the sensitization effect of Rh-WO_3_ nanosheets, we analyzed the oxygen adsorption behavior. Figure 5A shows a linear plot of sensor resistance (*R*_g_) with the partial pressure of oxygen (*P*_O2_) at a double logarithm-scale for pristine and 1wt.% Rh-WO_3_ sensors. It was observed that a linear relationship indicating a power-law response within all *P*_O2_ ranging from 0.06 to 0.99 atm (1 atm = 100% in volume) and the linear fitting coefficients were 0.42 and 0.62 for pristine and Rh-WO_3_, respectively. This indicated that the main type of oxygen adsorption was in the form of O^−^ for both sensors (Hua et al., 2018a,b) at working temperature of 300°C through:

(1) $$\begin{array}{l} {\text{O}}_{2}+2{\text{e}}^{-}\;⇄\;2{\text{O}}^{-} \end{array}$$

In case of 2 wt.% Rh-WO_3_, the linear plot of ln*R*_g_ with ln*P*_O2_ was also valid. Remarkably, the slope, i.e., fitting coefficient was just 0.29, considerably <0.5. However, it was unlikely that a large amount of Rh on the surface could tailor the form of oxygen ionisorption on the surface. The most probably explanation was that with increasing *P*_O2_ the oxidized state of Rh, which has been limited to be exposed to atmosphere due to the aggregation of particles, was enhanced and then the electronic interaction between Rh_2_O_3_ and WO_3_ surface was promoted. Consequently, new depletion regions formed, leading to an increase in sensor resistance with *P*_O2_ and a reduction in the fitting coefficient. This has also been observed in our previous Pt-WO_3_ sensor (Li et al., 2017).

According to our recent study, it was found that the power-law response of oxygen in the presence of reducing gas such as H_2_, CO, and acetone can be used to clarify the basic sensing mechanism of gas sensors (Hua et al., 2018c). Figure 5B shows the power-law response of oxygen in the presence of acetone (2 ppm) for pure and Rh-WO_3_ sensors. A very good linearity was observed for all sensors indicating that the basic sensing mechanism of acetone could be explained by the oxidation of acetone with oxygen adsorbates by:

(2) $$\begin{array}{l} \text{C}{\text{H}}_{3}\text{COC}{\text{H}}_{3}+8{\text{O}}^{-}\;\to \;3\text{C}{\text{O}}_{2}+{3\text{H}}_{2}\text{O}+8{\text{e}}^{-} \end{array}$$

For simplicity, it was assumed that acetone catalytic reaction was a complete reaction only producing CO_2_ and H_2_O. However, in fact the oxidation of acetone was rather complex. In addition, it was also found that linear coefficients of the power-law response were all around 0.5, which was consistent with Figure 5A and Equation (1). Importantly, for 2wt.%Rh-WO_3_, the fitting coefficient significantly raised up compared with that in the absence of acetone. This clearly supported our explanation for the degradation of sensitization effect with large loading amount of Rh and the reduction in the exponent of the power-law response to oxygen. In this respect, we believe that the basic sensitization mechanism of Rh on WO_3_ could be ascribed to the electronic interaction between Rh_2_O_3_ and WO_3_ (p-n junction), which was very similar with the fermi-level control model, popular for Pd-SnO_2_ sensors (Tang et al., 2015). The key factor to achieve a good sensitization effect highly relies on an elegant dispersion of Rh_2_O_3_ on WO_3_ surface, which can enhance the electronic interaction with WO_3_ surface as schematically drawn in Figure S6. This finding was similar with the case of Pt and Ru loaded WO_3_ nanosheets, however, it was significantly different with Pd and Fe loaded WO_3_. For the later one, the chemical sensitization effect of Pd and Fe plays a vital role through the reaction of surface lattice oxygens with reducing gases.

## Conclusion

In summary, Rh as a noble catalyst was dispersed onto the surface of WO_3_ nanosheets through a wet impregnation method. Experimental results indicated that Rh was in a form of oxidized state Rh_2_O_3_ on WO_3_ surface and an excessive amount of Rh can lead to an aggregation of Rh_2_O_3_ and poor sensitization effect as well. An electronic interaction between Rh_2_O_3_ and WO_3_ surface was evidenced by an extremely high argument in sensor resistance and it was thought that such an electronic was responsible for the observed sensitization effect of Rh loading. To achieve a good sensitization effect, an elegant dispersion of Rh_2_O_3_ is required, which highly relies on an effective dispersion method and a proper loading amount. Additionally, a power-law response to oxygen was observed for both pristine and Rh-WO_3_ in the presence of acetone, which indicts that oxygen adsorption on the surface of WO_3_ serves as a basic receptor function.

## Author contributions

YL performed the experiments and analyzed the data with help from DH, CT, CZ, and XT. ZH, MW and EL conceived and guided the study. ZQ wrote the manuscript based on experimental data.

### Conflict of interest statement

The authors declare that the research was conducted in the absence of any commercial or financial relationships that could be construed as a potential conflict of interest.
