# Supplementary material for: Acetone Sensing Properties and Mechanism of Rh-Loaded WO3 Nanosheets
Source: Front Chem. 2018 Sep 11;6:385. doi: 10.3389/fchem.2018.00385 (PMC6141622; doi:10.3389/fchem.2018.00385)
Supplement: Supplementary file 1 [file Table_1.DOCX]

**Supplementary materials for**

**Acetone sensing properties and mechanism of Rh-loaded WO_3_ nanosheets**

Zhilei Qiu, Zhongqiu Hua^[[1]](#footnote-1)^*, Yan Li, Mengjun Wang^[[2]](#footnote-2)^*, Dan Huang, Chen Tian, Chensheng Zhang, Xuemin Tian

Tianjin Key Laboratory of Electronic Materials and Devices, School of Electronics and Information Engineering, Hebei University of Technology, Tianjin 300401, China

**Supplementary material A**

The interaction of reducing gases and the surface redox process with WO_3_ and 1wt%Rh-WO_3_ were investigated by the TPR and the resistive response to oxygen. H_2_-TPR was performed in a flow of 10000 ppm H_2_ balanced with pristine N_2_ at a ramping rate of 15^o^C/min. Fig. S1 shows the procedure for pretreatment and measurement of TPR. The sample powders (0.05g, 30-50 mesh) were packed in a tubular quartz reactor and heat-treated at 400ºC for 30 min in a flow of air and then heat up to 500ºC at a speed of 15ºC/min. After cooling down to 50ºC in air and the TCD (thermal conductivity detector) was stabilized for 1h in 10000ppm H_2_ balanced with N_2_ before the TPR measurements.


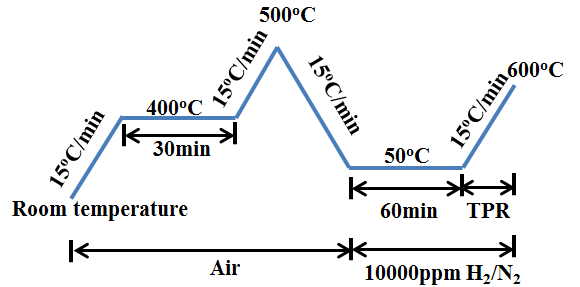


Fig. S1 The procedure of pretreatment and measurement of TPR.

**Supplementary material B**

Gas sensing tests were carried out by a conventional gas flow apparatus in Fig. S2. Four sensors were placed in a test chamber. The gas in the cylinder flowed into the mass flow controllers (MFC) through the gas line and entered the test chamber after mixing by the reversing valve. The gas flow rate was precisely controlled at a constant rate of 100 mL/min. The humidity of gas was less than 20 ppm and the temperature in the chamber was about 50ºC. At the same time, the oxygen concentrations was continuously measured by an oxygen analyzer. The data of sensors were collected by the data recorder. Finally, the tail gas treatment device is placed at the end of the device.


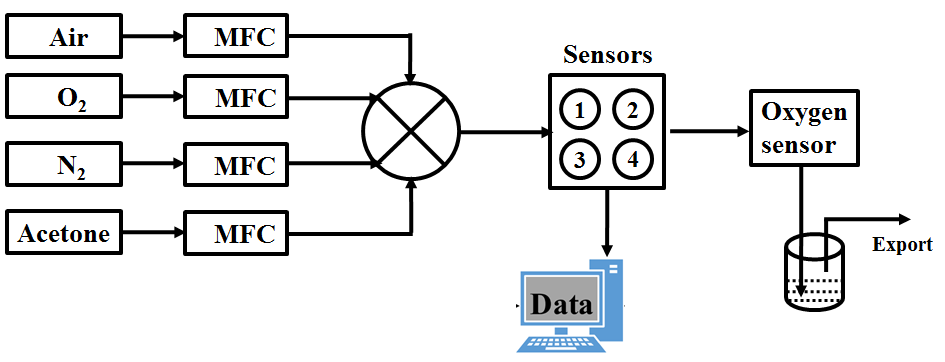


Fig. S2. Experimental set-up for the characterizations of gas sensing properties.

**Supplementary material C**

The XRD patterns demonstrated that the WO_3_ nanoparticles powders were in monoclinic phase (JCPDS: 43-1035) as shown in Fig. S3, and the mean crystalline size determined from the peaks of(200), (020) and (002) were 25.6 nm, 20.3 nm and 13.9 nm, respectively. However, peaks related to either metallic or oxidized state of Rh was not detected.

Fig. S3 XRD patterns of pristine WO_3_ and Rh-WO_3_ nanosheets powders

**Supplementary material D**

Fig. S4 presents the H_2_-TPR pattern of pristine and Pt-WO_3_ nanosheets powders. It is found that for pristine WO_3_ only one weak peak around 370ºC is observed within the sensor operation temperature, i.e. 400ºC Additionally, the intensity of hydrogen consumption is relatively small compared with Pt-WO_3_ for which, two peaks can be found. The first negative peak at low temperature is caused by desorption of H_2_ from metallic Pt.The second peak of Pt-WO_3_ exhibits a large consumption of H_2_, which is due to the reduction behavior of lattice oxygen of WO_3_. In contrary, such a large consumption cannot be observed in pristine WO_3_. Thus, it is proposed that Pt-loading greatly enhance the surface activity of the WO_3_ nanosheets，which could greatly promote the sensor response to reducing gases due to sensing reactions with lattice oxygen.

Fig.S4 H_2_-TPR pattern of WO_3_ and Pt-WO_3_ with a pretreatment in air atmosphere

**Supplementary material E**

Fig. S5 (a) the response of pristine WO_3_ and Rh-WO_3_ to 0.5 ppm acetone at different temperatures;(b) the resistance of pristine WO_3_ and Rh-WO_3_ as a function of temperatures in air; (c) and (d) is the test

of sensors stability at 350ºC

**Supplementary material F**


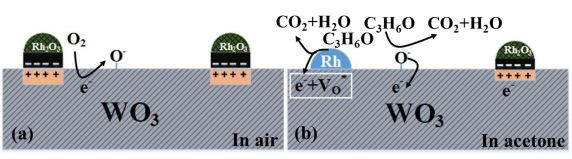


Fig. S6 Schematic diagram of the acetone sensing mechanism of (a) pristine WO_3_ and (b) Rh loaded-WO_3_

1. * Corresponding author: Zhongqiu. Hua, e-mail: zhongqiuhua@hebut.edu.cn, Tel.: +86 22 6043 8244; Fax: +86 22 6043 8244 [↑](#footnote-ref-1)
2. * Corresponding author: M.J. Wang, e-mail: wangmengjun@hebut.edu.cn, Tel.: +86 22 6043 8244; Fax: +86 22 6043 8244 [↑](#footnote-ref-2)
